# Safety and efficacy of manual vacuum aspiration under local anesthesia compared to general anesthesia in the surgical management of miscarriage: a retrospective cohort study

**DOI:** 10.1186/s13037-022-00328-7

**Published:** 2022-05-25

**Authors:** Toshiyuki Kakinuma, Kaoru Kakinuma, Ayaka Kaneko, Masataka Kagimoto, Yoshimasa Kawarai, Motomasa Ihara, Koyomi Saito, Yoshio Matsuda, Michitaka Ohwada, Hirokazu Tanaka, Nobuhiro Takeshima, Kaoru Yanagida

**Affiliations:** Department of Obstetrics and Gynecology, International University of Health and Welfare Hospital, 537-3 Iguchi, Nasushiobara, Tochigi 329-2763 Japan

**Keywords:** Miscarriage, Local anesthesia, Missed abortion, Obstetrical anesthesia, Pain, Vacuum aspiration

## Abstract

**Background:**

In Japan, dilatation & curettage (D&C) has been performed under general anesthesia as a surgery for an early pregnancy miscarriage for a long time. In 2016, manual vacuum aspiration (MVA) under general anesthesia was introduced at our hospital and has been used as a surgical treatment for first-trimester pregnancy miscarriage, with its utility to date being reported here. In July 2018, our hospital introduced the MVA procedure under local anesthesia. In this study, we evaluated the efficacy and safety of MVA under general and local anesthesia in first-trimester pregnancy miscarriage surgery in Japanese women.

**Methods:**

In this retrospective observational cohort study, we enrolled 322 pregnant women at less than 12 weeks of gestation, who underwent MVA surgery under local anesthesia (*n* = 166) or conventional general anesthesia (*n* = 156). The duration of surgery, blood loss volume, quantity of anesthesia, presence or absence of retained products of conception, and clinical complications were evaluated. In addition, the intraoperative pain and treatment satisfaction were assessed using the visual analog scale (VAS).

**Results:**

The duration of surgery was significantly shorter in the local anesthesia group. No significant differences were observed between both groups in terms of the blood loss volume and incidence of retained products of conception. In addition, no serious complications were observed in either group. No significant differences were noted between the two groups in the VAS scores for pain and treatment satisfaction.

**Conclusions:**

In this retrospective study, the use of MVA under local anesthesia for early pregnancy miscarriage surgery was found to be equally safe and effective when performed under conventional general anesthesia. This technique allowed the achievement of appropriate pain control with excellent patient satisfaction.

**Supplementary Information:**

The online version contains supplementary material available at 10.1186/s13037-022-00328-7.

## Background

In Japan, dilatation and curettage (D&C) under general anesthesia represents the preferred method for the surgical management of early pregnancy miscarriage [1, 2]. However, the World Health Organization (WHO), 2012 guidelines recommend manual vacuum aspiration (MVA) as a surgical procedure for miscarriage, particularly in first-trimester miscarriage [3]. MVA is a very safe method of abortion for pregnancies in the first trimester, and/or early second trimester up to 14 weeks of gestation [3].

MVA method has gained popularity and has already become a mainstream pregnancy miscarriage operation in Europe and United States. In October 2015, the Women’s MVA system was approved in Japan, and its use in the clinic has rapidly spread throughout Japan in recent years. In 2016, the MVA kit under general anesthesia was introduced at the hospital and has been used as a first-trimester pregnancy miscarriage surgical treatment, with its utility to date being reported [4]. The MVA kit is safe and effective and has a lower risk of endometrial damage, leading to a reduction of pain during and after the miscarriage surgery, thus simplifying anesthesia during the surgical procedure. The WHO recommends that all women, undergoing medical or surgical miscarriage, must be given analgesics as a standard procedure and routine general anesthesia is not recommended for MVA or D&C procedures [3].

In July 2018, the hospital introduced the MVA procedure, which was performed under local anesthesia. However, there is a paucity of studies evaluating the safety and efficacy of MVA kits under local anesthesia in the Japanese population. Therefore, in this study, our group aimed at investigating the efficacy and safety of MVA kits for pregnancy miscarriage surgical treatment under local and general anesthesia occurring during the first trimester in Japanese women.

## Methods

This retrospective cohort study was included pregnant patients at less than 12 weeks of gestation, who underwent a pregnancy miscarriage surgical treatment using an MVA kit between June 2016 and December 2020 at the International University of Health and Welfare Hospital in Tochigi, Japan. The study was approved by the Ethics Committee of the hospital (Approval number 13-B-323). All procedures were conducted following the ethical standards of the institutional and/or national research committee and the 1964 Helsinki Declaration and its later amendments or comparable ethical standards. All patients provided written and oral informed consent for the procedure and study participation after a proper explanation of the risks and benefits of the surgical procedure. Patients with contraindications to suspected ectopic pregnancy (no intrauterine pregnancy in the setting of complex adnexal mass or unilateral pain or ectopic pregnancy seen on ultrasonography), molar pregnancy, and completed abortion were excluded from this study.

The study participants were divided into two groups: the general anesthesia group that comprised 156 patients who underwent MVA surgery under conventional general anesthesia, and the local anesthesia group that comprised 166 patients who underwent MVA surgery under local anesthesia using paracervical blocks. For each group, the cervical canal was dilated the day before surgery using a cervical dilator (Lamicel®; Medtronic Japan, Inc., Tokyo, Japan). In the general anesthesia group, patients received intravenously 15 mg of pentazocine and 1–1.5 mg/kg of 1% propofol (Maruishi Pharmaceutical Co., Ltd., Osaka). Additional 10–20 mg of propofol was administered if patients complained of intraoperative pain during the surgical procedure. Paracervical canal blocks were used for the local anesthesia group. Using a 23-gage Cattelan needle (Terumo Corporation, Tokyo, Japan), 2.5 ml of 1% xylocaine (Nipro Corporation, Osaka) was locally injected to a depth of 15–20 mm at four different clock positions: 2, 4, 8, and 10 o’clock. The inner tube was pulled once to avoid erroneous intravascular administration and confirm no blood reflux occurred.

An MVA kit (Women’s MVA system) (Women’s Health Japan, Inc., Tokyo) was used for the pregnancy miscarriage surgical treatment. Following a sonde examination, the cervical canal was dilated to the diameter of the cannula equivalent to the gestational age (size of the gestational sac) using a dilator (a diameter of 6 mm for 5–7 weeks, 7 mm for 7–8 weeks, 8 mm for 8–9 weeks, and 9 mm for 9–12 weeks). The bicuspid valve of the aspirator was subsequently closed, the plunger was removed to create a vacuum within the syringe, and the uterine contents were aspirated after connecting with the inserted cannula. The pregnancy miscarriage surgical treatment was performed under guided transabdominal ultrasonography. After confirming the evacuation of the uterine contents, methylergometrine maleate injection (Aska Pharmaceutical Co., Ltd., Tokyo) was administered intramuscularly to complete the surgical procedure and all patients in both groups were administered orally methylergometrine maleate for 5 days postoperatively.

In both groups, we evaluated the duration of surgery, blood loss volume, presence or absence of retained products of conception (RPOC) and intraoperative and postoperative complications. In addition, we assessed the intraoperative pain and treatment satisfaction using the visual analog scale (VAS). Data regarding patient characteristics, preoperative clinical findings, and procedure complications were extracted from the patients’ medical records.

All results were expressed as mean ± standard deviation. For comparison between the two groups, the Student’s t-test and Fisher’s exact test were performed for continuous and categorical variables, respectively, and *p*-values of < 0.05 were considered statistically significant.

## Results

**Fig. 1:**
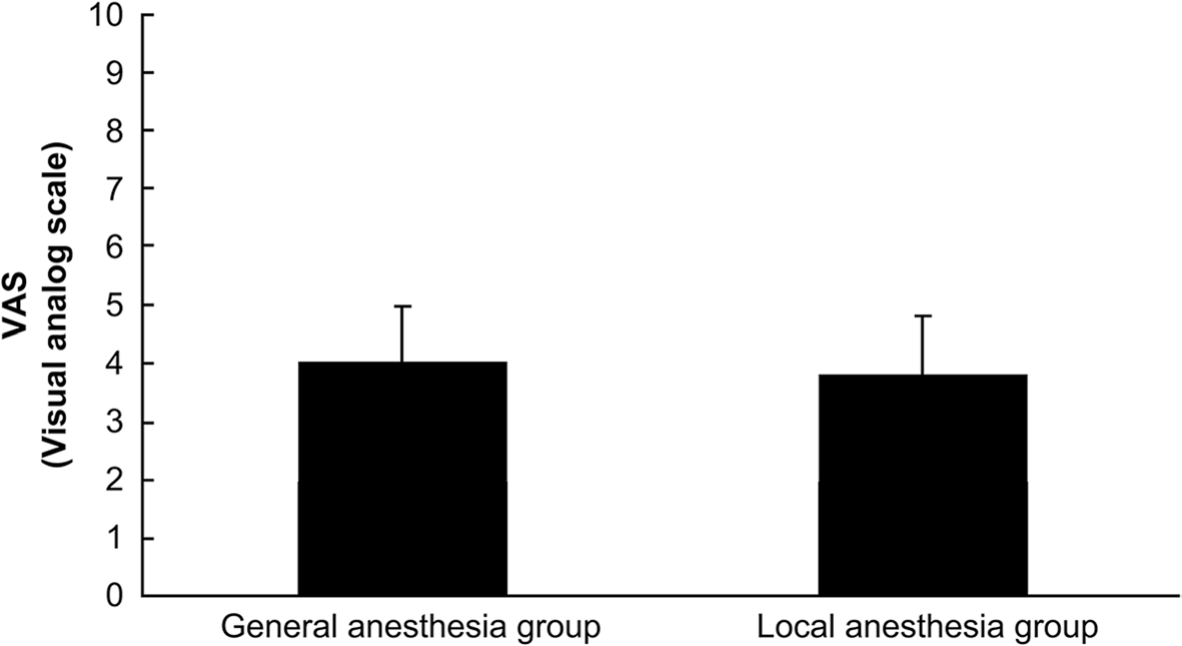
Surgical pain scale with manual vacuum aspiration under general anesthesia and local anesthesia. The visual analog scale pain score was 4.0 ± 1.8 and 3.8 ± 1.9 in the conventional general and local anesthesia group, respectively; there was no significant difference between the two groups (*p* = 0.347)

**Fig. 2:**
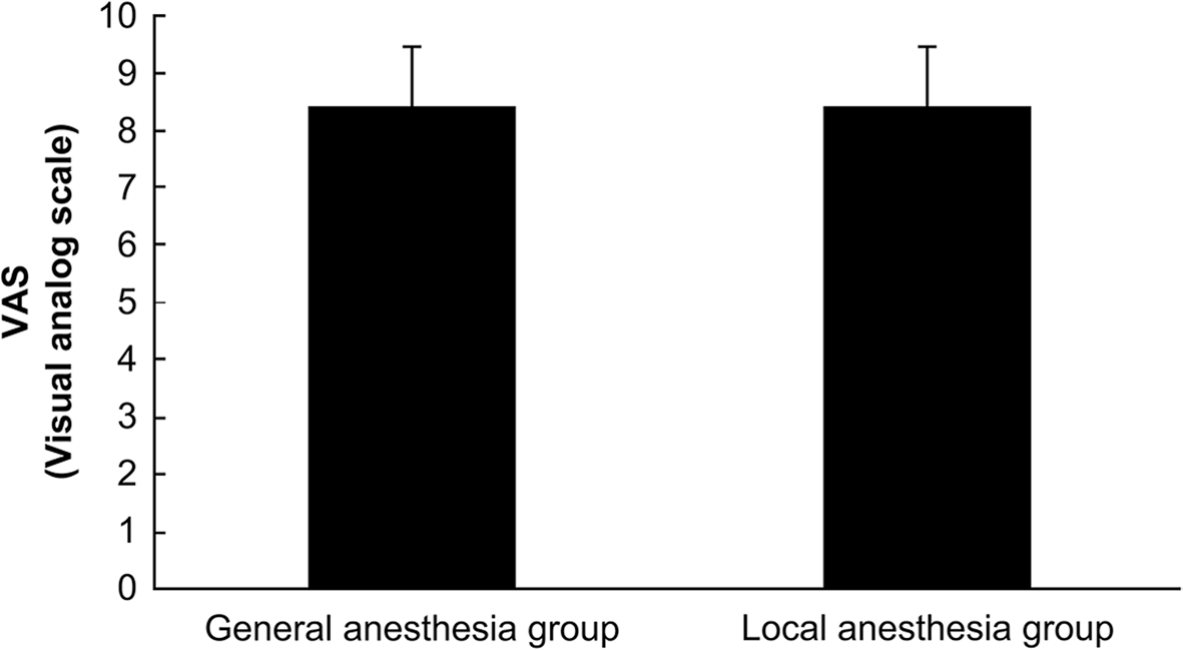
Treatment satisfaction with manual vacuum aspiration under general and local anesthesia. The visual analog scale score for treatment satisfaction was 8.5 ± 1.3 in the general anesthesia group and 8.5 ± 1.4 in the local anesthesia group; there was no significant difference between the two groups (*p* = 0.619)

Patient characteristics were compared between the two groups and are presented in Table 1. The mean age of the participant patients was 34.6 ± 6.1 years and 34.3 ± 5.5 years in the general and local anesthesia groups, respectively (*p* = 0.146); the gestational age was 8.6 ± 1.5 weeks in the general anesthesia group and 8.4 ± 1.3 weeks in the local anesthesia group; the gestational sac size was 30.5 ± 14.1 and 29.7 ± 12.8 mm in the general anesthesia group and the local anesthesia group, respectively (*p* = 0.360); these results showed no significant differences in the pregnancy history between the two groups (Table 1). The duration of surgery was 5.8 ± 2.4 and 4.7 ± 2.1 min in the general anesthesia and local anesthesia groups, respectively (*p* = 0.004), and it was significantly shorter in the local anesthesia group (Table 2). In the general anesthesia group, only one patient (0.6%) had RPOC and a blood loss volume of at least 100 ml, and there were no such cases observed in the local anesthesia group. There were no complications from anesthesia in either group (Table 2). The VAS score for intraoperative pain (Fig. 1) was 4.0 ± 1.8 and 3.8 ± 1.9 in the general anesthesia group and the local anesthesia group, respectively (*p* = 0.347), and the VAS score for treatment satisfaction was 8.5 ± 1.3 in the general anesthesia group and 8.5 ± 1.4 in the local anesthesia group (*p* = 0.619) (Fig. 2). The VAS scores data did not show any significant differences for intraoperative pain and treatment satisfaction between the two groups.

**Table 1:** Patient characteristics

	General anesthesia group (*n* = 156)	Local anesthesia group (*n* = 166)	*p*-value
Average age (years)	34.6 ± 6.1	34.2 ± 5.5	0.146
Gravida	2.2 ± 1.4	2.2 ± 1.3	0.469
Para	1.0 ± 0.8	0.9 ± 0.9	0.277
Gestational age (weeks)	8.5 ± 1.5	8.4 ± 1.8	0.281
Size of gestational sac (mm)	30.5 ± 14.1	29.7 ± 12.8	0.360
Mean ± standard deviation

**Table 2:** Surgical performance

	General anesthesia group (*n* = 156)	Local anesthesia group (*n* = 166)	*p*-value
Duration of surgery (min)	5.8 ± 2.4	4.7 ± 2.1	0.004
Bleeding of ≥ 100 ml (cases)	1	0	-
Retention of uterine contents (cases)	1	0	-

## Discussion

On the basis of the WHO recommendations, and with support from the United States Agency for International Development, the Ipas MVA was developed and widely used in the clinic across a large number of countries worldwide. In Japan, D&C is still performed under general anesthesia as a type of pregnancy miscarriage surgical treatment and in more than 50% cases [2]. In October 2015, however, the Women’s MVA system was approved for use as a surgical treatment for first-trimester miscarriages in routine clinical practice and is now gaining popularity in Japanese medical institutions.

Asherman’s syndrome consists of partial or widespread intrauterine adhesions with consequent menstrual abnormalities, infertility, implantation failure (despite assisted reproduction), and placental abnormalities [5]. It occurs when the endometrium sustains trauma or ischemia. If this damage extends beyond the functional endometrial layer to the basal layer, the endometrial epithelium fails to regenerate, causing endometrial surface adhesions. In several cases, this endometrial damage occurs due to surgical miscarriage [5]. The partial or total absence of endometrial tissue and the resulting Asherman’s syndrome precludes normal endometrial thickening during the menstrual cycle, causing uterine amenorrhea or uterine infertility. MVA, as a method of surgical management of miscarriage, is useful because it protects the endometrium and reduces the occurrence of Asherman’s syndrome. In a survey conducted by Gilman et al. in patients who underwent treatment for first-trimester miscarriage, electric vacuum aspiration and D&C were risk factors for Asherman’s syndrome, but MVA was not a risk factor (*P* = 0.003) [6]. Moreover, it has been shown that the pregnancy rate is very low in patients with endometrial thinning [7], and obviously, suggesting that endometrial growth is an important factor for implantation and pregnancy. In another study, Azumaguchi et al. [8] found a clear inverse relationship between endometrial thickness and previous D&C rates, suggesting that D&C is the primary cause of endometrial thinning [8]. In an evaluation performed before and after miscarriage surgery, our observation confirmed that there was no endometrial thinning when MVA was used [4]. MVA has a limited risk of endometrial damage and reduces the occurrence of Asherman’s syndrome and adenomyosis. Therefore, MVA is considered effective for endometrial protection and it is a surgical procedure that allows surgery without intrauterine curettage, which eliminates the need for sterile surgical tools and reduces the risk of infection Furthermore, the plastic cannulas used in MVA have a low potential of damaging the endometrial basal layer due to their suitable hardness and high flexibility.

The low risk of endometrial damage diminished the pain during and after miscarriage surgery, thereby allowing the simplification of anesthesia administered during surgery. The WHO recommends that all women, treated with drug-induced and surgical miscarriage, receive analgesics as standard care; however, routine general anesthesia is not recommended [3]. Compared with conventional D&C, MVA allows abortion surgery to be performed by only slightly dilating the cervix using a thin cannula. As previously described, MVA can manage pain easier from the perspective of endometrial protection and is associated with a shorter duration of surgery, which may lead to pain relief during and after surgery. Avoiding general anesthesia can also shorten the recovery time, obviate the need for equipment and personnel to perform general anesthesia, and reduce the risk of anesthesia-related complications, thus leading to cost savings. Although there are numerous advantages of the local anesthesia method, the risk of local anesthetic poisoning must always be kept in mind. Attention toward the prevention of local anesthesia overdoses or improper administration in the blood vessel is required. During the procedure, an electrocardiogram, sphygmomanometer and pulse oximeter are attached to the patient, and the surgery is performed by securing an intravenous route. Additionally, an emergency system is put together, such as the preparation of oxygen bags, masks, and emergency drugs, enabling appropriate measures including cardiopulmonary resuscitation in the event of serious complications.

In this retrospective study, MVA miscarriage surgery could be safely performed under local and conventional general anesthesia in the first trimester of pregnancy. Pain control during miscarriage surgery was favorable, and the patients obtained a high degree of treatment satisfaction. Although pregnancy miscarriage surgical treatment have been performed for a long time under general anesthesia in Japan, MVA under local anesthesia will be accepted as a surgical miscarriage method during the first trimester of pregnancy for Japanese women in the future. As part of pre-conception care, there is a need for establishing low invasion intrauterine exenteration for the next pregnancy without creating new sterility factors, especially in cases desire to bear children.

## Conclusions

The surgical management of first-trimester miscarriage via MVA was shown to be safe and effective, similar to the surgical treatment under general anesthesia. Hence, MVA miscarriage surgery performed under local anesthesia represents a feasible alternative option for the surgical management of first-trimester miscarriages in Japanese women.

## Supplementary Information

**Additional file 1.**

## Data Availability

The datasets used and/or analyzed during the current study are available from the corresponding author on reasonable request.

## Abbreviations

MVA: Manual vacuum aspiration
RPOC: Retained products of conception
VAS: Visual analog scale
WHO: World Health Organization
